# Expression relationship and significance of NEAT1 and miR-27a-3p in serum and cerebrospinal fluid of patients with Alzheimer’s disease

**DOI:** 10.1186/s12883-022-02728-9

**Published:** 2022-06-03

**Authors:** Lijie He, Zongnan Chen, Jing Wang, Heqiang Feng

**Affiliations:** 1 Department of Clinical Laboratory, Tianjin Fifth Central Hospital, 41 Zhejiang Road, Tianjin, 300450 People’s Republic of China; 2 General Surgery, Tianjin Fifth Central Hospital, Tianjin, 300450 People’s Republic of China

**Keywords:** Alzheimer’s disease, Long chain non-coding RNA nuclear-enriched abundant transcript 1, miR-27a-3p, Amyloid β, Correlation

## Abstract

**Objective:**

To explore the expression relationship and significance of long chain non-coding RNA nuclear-enriched abundant transcript 1 (LncRNA NEAT1) and miR-27a-3p in serum and cerebrospinal fluid of patients with Alzheimer’s disease (AD).

**Methods:**

Sixty-six AD patients received by the Department of Neurology of our hospital from October 2019 to September 2021 were gathered, according to the Clinical Dementia Rating Scale (CDR) score, they were grouped into mild group (≤1 point, *n* = 41) and moderate-to-severe group (> 1 point, *n* = 25). Another 32 cases of serum and cerebrospinal fluid samples from outpatient physical examination personnel were regarded as the control group. The general materials on all subjects was recorded and cognition was assessed;real-time quantitative PCR was performed to measure the expression levels of miR-27a-3p and NEAT1 in serum and cerebrospinal fluid;enzyme-linked immunosorbent assay was performed to measure the protein levels of β-amyloid precursor protein cleaving enzyme 1 (BACE1), β-amyloid (Aβ) 40 and Aβ42 in cerebrospinal fluid;Spearman’s method was performed to analyze the correlation of serum miR-27a-3p and NEAT1 levels with MMSE and MoCA scores;Pearson method was performed to analyze the correlation between serum miR-27a-3p and NEAT1 levels and Aβ deposition standard uptake value ratio (SUVR) and cerebrospinal fluid miR-27a-3p, NEAT1, BACE1, Aβ42 and Aβ40 levels.

**Results:**

The MMSE score, MoCA score, serum miR-27a-3p level, cerebrospinal fluid miR-27a-3p, Aβ42 levels and Aβ42/Aβ40 ratio of AD patients in mild group and moderate-to-severe group were all lower than those in the control group, and the moderate-to-severe group were lower than the mild group (all *P* < 0.05);the serum NEAT1 level, SUVR, and cerebrospinal fluid NEAT1 and BACE1 levels were higher than those in the control group, and the moderate-to-severe group were higher than the mild group (all *P* < 0.05). Serum NEAT1 level in AD patients was positively correlated with SUVR, cerebrospinal fluid NEAT1 and BACE1 (*r* = 0.350, 0.606, 0.341, all *P* < 0.05);serum miR-27a-3p level was positively correlated with cerebrospinal fluid miR-27a-3p level (*r* = 0.695, *P* < 0.05), and negatively correlated with SUVR and cerebrospinal fluid BACE1 level (*r* = − 0.521, − 0.447, both *P* < 0.05).

**Conclusions:**

The expression trends of NEAT1 and miR-27a-3p in the serum and cerebrospinal fluid of AD patients are consistent, the level of NEAT1 is increased, and the level of miR-27a-3p is decreased. The levels of the two are negatively correlated, which is related to the degree of Aβ deposition in the brain of AD patients and is involved in the progression of AD.

## Introduction

Alzheimer’s disease (AD) is a common degenerative neurological disease which is mainly memory impairment and cognitive impairment and the aging incidence rate of AD is increasing year by year [1, 2]. Meanwhile, research has shown that amyloid β peptides (Aβ1–40 and Aβ1–42) and hyperphosphorylation of tau protein (p-Tau) contribute to AD development. Amyloid β plaques and neurofibrillary tangles are the main pathological features of AD patients and there is a lack of effective clinical treatment [3, 4]. Therefore, the study of its pathogenesis may be conducive to the development of effective diagnosis and treatment measures.

Nuclear-enriched abundant transcript 1 (NEAT1) is a long chain non-coding RNA (LncRNA) recently found to be closely related to the progress of AD. Previous studies have shown that lncRNA NEAT1 is involved in regulating the development and progression of cervical cancer, pancreatic cancer, liver cancer, prostate cancer and multiple myeloma [5–9]. Micro-27a is located on human chromosome 19 and it is sheared to form miR-27a-3p. miR-27a-3p plays an important role in glioma, pulmonary fibrosis and other diseases [10–13]. Previous studies showed that the relative fluorescence intensity decreased when micro-27a-3p mimic was co-transfected with NEAT1 wild type. Micro-27a-3p could be enriched on lncRNA NEAT1. The studies indicated that lncRNA NEAT1 could regulate micro-27a-3p through predictive sites. Cell experiments confirmed that NEAT1 can sponge miR-107, miR-124 and enhance Aβ deposition, aggravating Aβ induced neuronal injury [14, 15]. This study predicted that miR-27a-3p was also the target gene of NEAT1 through Starbase website, and the expression of miR-27a-3p was down regulated in cerebrospinal fluid of AD patients [16]. The study found that the expression of NEAT1 was up-regulated in AD cells and animal models, and the expression of miR-27a-3p was down regulated. NEAT1 may regulate the development of AD by down regulating miR-27a-3p [17], but the expression relationship and clinical significance of NEAT1 in serum and cerebrospinal fluid of AD patients were unclear. Therefore, this observational study analyzed the relationship between the expression of NEAT1 and miR-27a-3p in serum and cerebrospinal fluid of AD patients, and analyzed the relationship between the levels of NEAT1 and miR-27a-3p in serum and cerebrospinal fluid Aβ level and cognitive level in order to explore their role in AD.

## Materials and methods

### General materials

Sixty-six patients with AD treated in the Department of Neurology of our hospital from October 2019 to September 2021 were collected. All participants met NIA-AA, 2011 edition of AD core diagnostic criteria [18]. The main complaint of participants was cognitive impairment and confirmed by the accompanying person. The participants age from 60 to 80 years old. All participants assigned informed consent and could cooperate with the investigator. Exclusion criteria for patients who have history traumatic brain injury, stroke, epilepsy and other neurological diseases and even have mental diseases (history) such as delirium, schizophrenia and depression, Unable to complete the score of cognitive scale, combined with brain tumor and abnormal thyroid function, there are contraindications to positron emission computed tomography (PET) or lumbar puncture. Forty-one cases were divided into mild group (≤1 point) and 25 cases were divided into moderate to severe group (> 1 point) according to the score of clinical dementia rating (CDR). In addition, 32 subjects whose age and gender were matched with AD patients and whose cognitive evaluation was normal were collected. This study was approved by the hospital ethics committee and all materials collection and sample testing obtained the informed consent of patients and their families, and signed the informed consent form.

### Methods

#### General materials collection

The materials of age, gender, education, alcohol consumption, smoking, hypertension, diabetes, neuropathy or mental illness and imaging data were collected by using medical records. The A β standardized uptake value ratio (SUVR) in brain was calculated based on C^11^-PIB PET imaging and which was the represent A β in the brain degree of deposition.

#### Evaluation of cognitive scale

All participants were assessed with mini-mental state examination (MMSE), Montreal Cognitive Assessment (MoCA) and CDR, among which CDR was completed by professional evaluators in our hospital.

#### Detection NEAT1 and miR-27a-3p in the serum

Five milliliter of fasting elbow venous blood of all participants were collected in the morning and placed in a 15 ml centrifuge tube at room temperature for 1 h (within 2 h) and the serum was centrifuged at 2000 rmp/min (effective centrifugation radius 10 cm). Total RNA in serum samples was extracted with RNA Extraction Reagent (Invitrogen Company, China) and reverse transcribed into cDNA with miRNA or LncRNA cDNA first strand synthesis Kit (Tiangen Biotech (Beijing) Co., Ltd). Then samples were prepared according to the instructions of the corresponding real-time fluorescence quantitative PCR premix Kit (Tiangen Biotech (Beijing) Co., Ltd) to 20 μL PCR reaction system and that was put into real-time fluorescence quantitative PCR instrument (BioRad Company, China)) to amplify miR-27a-3p, NEAT1 and their internal parameters U6 and GAPDH. After the reaction, collect each Ct value and the relative expressions of miR-27a-3p and NEAT1 were calculated by 2^-ΔΔCT^ method. U6, forward 5 ‘- CTCGCTTCGGCAGCACA-3’ and reverse5 ‘- AACGCTTCACGAATTGCG-3’; miR-27a-3p, forward 5 ‘- GGGTTCACAGTGGCTAAG-3’ and reverse 5 ‘- CAGTGGTCGTGGAGT-3’; GAPDH, forward 5 ‘- GTCGGTGAACGGATTTGG-3’ and reverse 5 ‘- TCCCGTTGATGACCAGTTC-3’; NEAT1, forward 5 ‘- GGCAGGTCTAGTTGGGCAT-3’ and reverse 5 ‘- CCTCATCCCCTCCCAGTACA-3’.

#### Assessment of the studied parameters in the cerebrospinal fluid

In the early morning 8:00–10:00, 5 ml of fasting cerebrospinal fluid of all participants were collected through lumbar puncture and centrifuged in a 15 ml centrifuge tube, immediately after collection (no more than 1 hour) to the laboratory. The expression levels of miR-27a-3p and NEAT1 in cerebrospinal fluid were detected by real-time fluorescence quantitative PCR, Total RNA in serum samples was extracted with RNA Extraction Reagent (Invitrogen Company, China) and reverse transcribed into cDNA with miRNA or LncRNA cDNA first strand synthesis Kit (Tiangen Biotech (Beijing) Co., Ltd). Then samples were prepared according to the instructions of the corresponding real-time fluorescence quantitative PCR premix Kit (Tiangen Biotech (Beijing) Co., Ltd) to 20 μL PCR reaction system and that was put into real-time fluorescence quantitative PCR instrument (BioRad Company, China)) to amplify miR-27a-3p, NEAT1 and their internal parameters U6 and GAPDH. After the reaction, collect each Ct value and the relative expressions of miR-27a-3p and NEAT1 were calculated by 2^-ΔΔCT^ method. U6, forward 5 ‘- CTCGCTTCGGCAGCACA-3’ and reverse5 ‘- AACGCTTCACGAATTGCG-3’; miR-27a-3p, forward 5 ‘- GGGTTCACAGTGGCTAAG-3’ and reverse 5 ‘- CAGTGGTCGTGGAGT-3’; GAPDH, forward 5 ‘- GTCGGTGAACGGATTTGG-3’ and reverse 5 ‘- TCCCGTTGATGACCAGTTC-3’; NEAT1, forward 5 ‘- GGCAGGTCTAGTTGGGCAT-3’ and reverse 5 ‘- CCTCATCCCCTCCCAGTACA-3’. Measured the concentration of the analytes in cerebrospinal fluid were detected by enzyme-linked immunosorbent assay β- site amyloid precursor protein cleaving enzyme 1 (BACE1, ELISA kit of Abcam, catalogue number: EPR22802–143, China), Aβ 40 and Aβ 42 protein (Aβ Test kit of INNOTEST®, CanAg Diagnostic (Beijing)Co., Ltd.) content.

### Statistical analysis

SPSS 25.0 statistical software was used for statistical analysis. If the measurement data conform to the normal distribution, it was expressed as the mean ± standard deviation ($\overline{x}$ ±s). The multi group comparison adopted one-way ANOVA, and the pairwise comparison adopted LSD (least significant difference)-*t* test; If it did not conform to the normal distribution, it was represented by M (*P*25, *P*75) and analyzed by the Kruskal–Wallis test for multi group comparison, and all pairwise method was used for pairwise comparison. The counting data were expressed in cases (%), and the comparison between groups was adopted χ ^2^ inspection. The correlation between serum NEAT1, miR-27a-3p and MMSE and MOCA scores was determined by Spearman method; Pearson method was used for other correlation analysis. *P* < 0.05 was statistically significant.

## Results

### Comparison of general materials between AD patients and control group

There were no significant differences in age, gender, education level, alcohol consumption, smoking, hypertension, and diabetes among the mild and moderate to severe group AD patients and the control group (*P* > 0.05). MMSE scores and MOCA scores of AD patients in mild group and moderate to severe group were lower than those in the control group and AD patients in moderate to severe group were lower than those in mild group (all *P* < 0.05), as shown in Table 1.

**Table 1:** Comparison of general materials between AD patients and control group

Group	Cases	Age (Years, ^−^*X ± S*)	Gender (Male/Female, Cases)	Education Level (Years, ^−^*X ± S*)	Alcohol consumption (Cases, %)	Smoking (Cases, %)	Hypertension (Cases, %)	Diabetes (Cases, %)	MMSE score (score, *M*(*P25, P75*))	MoCA score (score, *M*(*P25, P75*))
Control group	32	69.45 ± 7.36	18/14	7.65 ± 2.13	5(15.63)	14 (43.75)	11 (34.38)	6 (18.75)	27 (26, 29.75)	27 (26, 28)
Mild group	41	71.31 ± 7.52	23/18	7.98 ± 2.26	9 (21.95)	19 (46.34)	13 (31.71)	7 (17.07)	20 (18, 21)^#^	17 (13, 19)^#^
Moderate to severe group	25	72.57 ± 6.83	15/10	6.92 ± 2.05	4 (16.00)	13 (52.00)	12 (48.00)	6 (24.00)	9 (8, 12)^#*^	9 (6.5, 11)^#*^
*F/χ*^*2*^*/H* value	–	1.336	0.112	1.871	0.909	0.383	1.888	0.489	85.197	82.854
*P* value	–	0.268	0.946	0.160	0.645	0.826	0.389	0.738	0.000	0.000

*MMSE* Mini-mental state examination, *MoCA* Montreal cognitive assessment, *MoCA* Montreal cognitive assessment

Compared with the control group, ^#^ *P* < 0.05; Compared with mild group, * *P* < 0.05

### Comparison of serum NEAT1 and miR-27a-3p levels between AD patients and control group

The level of serum NEAT1 in mild and moderate to severe AD patients was higher than that in the control group, and the level of serum NEAT1 in moderate to severe AD patients was higher than that in mild AD patients (*P* < 0.05); The level of serum miR-27a-3p was lower than that of the control group, and the level of moderate to severe AD patients was lower than that of mild AD patients (*P* < 0.05), as shown in Table 2.

**Table 2:** Comparison of serum NEAT1 and miR-27a-3p levels between AD patients and control group (^*−*^*x ± s*)

Group	Cases	NEAT1/GAPDH	miR-27a-3p/U6
Control group	32	1.05 ± 0.20	0.97 ± 0.22
Mild group	41	2.31 ± 0.64^#^	0.55 ± 0.13^#^
Moderate to severe group	25	3.13 ± 0.76^#*^	0.46 ± 0.06^#*^
*F* value	–	96.049	93.850
*P* value	–	0.000	0.000

*NEAT1* Nuclear-enriched abundant transcript 1

Compared with the control group, ^#^*P* < 0.05; Compared with mild group, ^*^ *P* < 0.05

### The comparison SUVR, cerebrospinal fluid NEAT1, miR-27a-3p, BACE1, Aβ 42 and Aβ 40 level in AD patients and control group

The levels of SUVR, NEAT1 and BACE1 in cerebrospinal fluid of AD patients in mild group and moderate to severe group were higher than those in the control group, and the levels of AD patients in moderate to severe group were higher than those in mild group (*P* < 0.05); Cerebrospinal fluid miR-27a-3p, Aβ 42 level and Aβ 42/ Aβ 40 ratio were lower than that of the control group, and the AD patients in the moderate to severe group were lower than those in the mild group (all *P* < 0.05), as shown in Table 3.

**Table 3:** The comparison SUVR, cerebrospinal fluid NEAT1, miR-27a-3p, BACE1, Aβ 42 and Aβ 40 level in AD patients and control group (^−^*x ± s*)

Group	Cases	NEAT1/GAPDH	miR-27a-3p/U6	BACE1(ng/mL)	Aβ 40(pg/mL)	Aβ 42(pg/mL)	SUVR	Aβ 42/ Aβ 40
Control group	32	1.01 ± 0.23	1.03 ± 0.31	21.39 ± 3.73	10,708.99 ± 1979.17	499.99 ± 53.63	0.74 ± 0.15	0.048 ± 0.010
Mild group	41	3.51 ± 1.24^#^	0.48 ± 0.10^#^	55.78 ± 5.98^#^	10,477.95 ± 2192.92^#^	303.55 ± 36.77^#^	1.50 ± 0.29^#^	0.030 ± 0.008^#^
Moderate to severe group	25	4.30 ± 1.65^#*^	0.35 ± 0.10^#*^	72.32 ± 16.08^#*^	11,019.63 ± 2807.62^#*^	231.45 ± 34.14^#*^	1.76 ± 0.52^#*^	0.022 ± 0.007^#*^
*F* value	–	65.263	102.897	234.697	0.432	321.684	75.791	73.370
*P* value	–	0.000	0.000	0.000	0.651	0.000	0.000	0.000

*NEAT1* Nuclear-enriched abundant transcript 1, *Aβ* Amyloid β, *SUVR* Standardized uptake value ratio, *BACE1* β-site amyloid precursor protein cleaving enzyme 1

Compared with the control group, ^#^*P* < 0.05; Compared with mild group, ^*^ *P* < 0.05

### The correlation of serum NEAT1 with SUVR, cognitive function score, cerebrospinal fluid NEAT1, BACE1, Aβ 42 and Aβ 40 level in patients with AD

The level of serum NEAT1 in AD patients was positively correlated with SUVR, NEAT1, BACE1 in cerebrospinal fluid (*r* = 0.350, 0.606 and 0.341, *P* < 0.05). There was no correlation between level Aβ 42、Aβ40, MMSE score and MOCA score (*P* > 0.05), as shown in Table 4.

**Table 4:** The correlation of serum NEAT1 with SUVR, cognitive function score, cerebrospinal fluid NEAT1, BACE1, Aβ 42 and Aβ 40 level in patients with AD

Index		Cerebrospinal fluid						
		SUVR	NEAT1	BACE1	Aβ 42	Aβ 40	MMSE score	MoCA score
Serum NEAT1	*r* value	0.350	0.606	0.341	−0.096	−0.115	−0.118	−0.220
	*P* value	0.000	0.000	0.005	0.445	0.358	0.343	0.076

*NEAT1* Nuclear-enriched abundant transcript 1, *Aβ* Amyloid β, *SUVR* Standardized uptake value ratio, *BACE1* β-site amyloid precursor protein cleaving enzyme 1, *MMSE* Mini-mental state examination, *MoCA* Montreal cognitive assessment

### The correlation of serum miR-27a-3p to SUVR, cognitive function score, cerebrospinal fluid miR-27a-3p, BACE1, Aβ 42 and Aβ 40 level in patients with AD

The level of serum miR-27a-3p in AD patients was positively correlated with the level of cerebrospinal fluid miR-27a-3p (*r* = 0.695, *P* < 0.05), negatively correlated with the level of SUVR and cerebrospinal fluid BACE1 (*r* = − 0.521, − 0.447, *P* < 0.05), There was no correlation between level Aβ 42、Aβ40, MMSE score and MOCA score (*P* > 0.05), as shown in Table 5.

**Table 5:** The correlation of serum miR-27a-3p to SUVR, cognitive function score, cerebrospinal fluid miR-27a-3p, BACE1, Aβ 42 and Aβ 40 level in patients with AD

Index		Cerebrospinal fluid						
		SUVR	miR-27a-3p	BACE1	Aβ 42	Aβ 40	MMSE score	MoCA score
Serum miR-27a-3p	*r* value	− 0.521	0.695	−0.447	0.074	−0.047	0.1151	0.144
	*P* value	0.000	0.000	0.000	0.554	0.710	0.225	0.247

*Aβ* Amyloid β, *SUVR* Standardized uptake value ratio, *BACE1* β-site amyloid precursor protein cleaving enzyme 1, *MMSE* Mini-mental state examination, *MoCA* Montreal cognitive assessment

### Correlation between serum and cerebrospinal fluid NEAT1 and miR-27a-3p in patients with AD

**Fig. 1:**
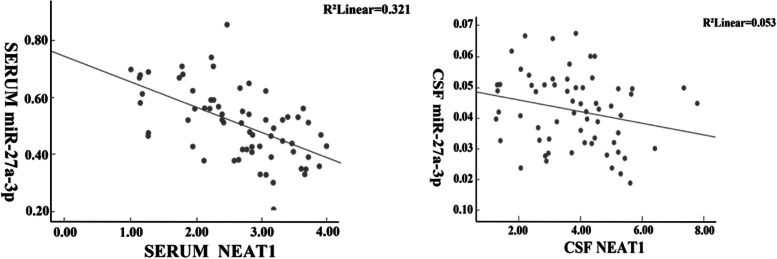
Correlation analysis between serum (left) and cerebrospinal fluid (right) NEAT1 and miR-27a-3p

The levels of NEAT1 in serum and cerebrospinal fluid of AD patients were negatively correlated with the levels of miR-27a-3p (r_serum_ = − 0.567, r_cerebrospinal fluid_ = − 0.347, *P* < 0.05), as shown in Fig. 1.

## Discussion

AD is a common degenerative neurological disease. With the progress of the disease, patients’ cognition and living ability gradually decline, and the burden of society and family also increases. Therefore, it is necessary to screen key populations and treat them as soon as possible [19]. Recent studies have shown that non-coding RNAs such as miRNA and LncRNA were involved in the occurrence and development of AD. For example, the down-regulation of miR-132 was the key cause of neuronal death in the early stage of AD [20, 21], and miR-132 supplement could promote neurite growth and branching by reducing the pathological modification of tau protein [22] to protect neurons. BACE1-antisense transcripts are up-regulated in the brain of AD patients, which could stabilize BACE1 mRNA by binding and promote its increased expression and Aβ production and plaque formation [23]. In addition, LncRNA could also be used as endogenous competitive RNA to adsorb and bind miRNA to increase the expression of miRNA target mRNA. For example, LncRNA XIST could regulate the expression of BACE1 by adsorbing miR-124, and then affect Aβ produce [24].

Previous studies have found that NEAT1 was highly expressed in the brain of AD rats, which was related to Aβ sedimentation [15, 17]. Sala Frigerio et al. [16] found that miR-27a-3p was low expressed in cerebrospinal fluid of AD patients. In our study, high expression of NEAT1 and low expression of miR-27a-3p were detected in serum and cerebrospinal fluid of AD patients, and the levels of serum NEAT1 and miR-27a-3p were positively correlated with the levels of cerebrospinal fluid NEAT1 and miR-27a-3p respectively, which indicating that the expression trend of NEAT1 and miR-27a-3p in peripheral circulation and cerebrospinal fluid was consistent, and the levels of serum NEAT1 and miR-27a-3p may reflect their expression in cerebrospinal fluid. This study also found that serum NEAT1 levels were positively correlated with SUVR and cerebrospinal fluid BACE1 levels, while serum miR-27a-3p levels were negatively correlated with SUVR and cerebrospinal fluid BACE1 levels, which indicating that serum NEAT1 and miR-27a-3p levels could reflect the Aβ degree of sedimentation in the brain of patients with AD.

Scientific data show that LncRNAs play an important role in the pathogenesis of Parkinson’s disease (PD). Recent studies have focused on a new class of non-coding RNA, These circRNAs are now recognized as having important biological roles, For example, ciRS-7 it has been identified as a sponge for miR-7, of note, high neuronal α-synuclein expression is implicated in PD, and SNCA is a target gene of miR-7. Another circRNA, circSLC8A1, was found to increase in the substantia nigra of PD patients, and this circRNA carrier sites for miR-128, an abundant miRNA that regulates neuronal excitability. Previous studies have shown that lncRNA NEAT1 is involved in regulating the development and progression of cervical cancer, pancreatic cancer, liver cancer, prostate cancer and multiple myeloma [5–9], NEAT1 and miR-27a-3p have not been reported in PD and other neurodegenerative diseases, it was speculated that NEAT1 and miR-27a-3p are specific to AD.

Previous studies have found that NEAT1 could target adsorbed miRNA and regulate BACE1 expression [14, 15]. This study also found that the levels of NEAT1 in serum and cerebrospinal fluid were negatively correlated with the levels of miR-27a-3p, and the website shown that there were binding sites between NEAT1 and miR-27a-3p, and there were also binding sites between BACE1 and miR-27a-3p. It was speculated that NEAT1 may target miR-27a-3p to regulate BACE1 expression. BACE1 was the key enzyme synthesized to Aβ and has become an important target for drug treatment of AD [25, 26]. It is speculated that NEAT1 was highly expressed in the brain of AD patients and could maintain the stability of BACE1 and promote its expression by adsorbing miR-27a-3p, so as to promote the Aβ production of and sedimentation into spots in the brain, resulting in neuronal degeneration and damage [17, 27]. However, the expression trend of NEAT1 and miR-27a-3p in the brain tissue of AD patients remained to be analyzed. It has been reported that small molecule drugs targeting non-coding RNA were expected to be used in the treatment of AD and other diseases [28]. It was speculated that drugs targeting NEAT1 or miR-27a-3p may be used in the treatment of AD. Subsequently, this study also analyzed the correlation between serum NEAT1 level and cognitive scale score, but the correlation between cerebrospinal fluid NEAT1 and miR-27a-3p level with MMSE score and MOCA score was not observed in AD patients. On the one hand, we speculated that may be related to the small sample size of our study, on the other hand, it may be related to the MMSE score and MOCA score for the impact relatively large for course of disease, education, and the sample size will be expanded for further analysis in the future.

To sum up, the expression trends of NEAT1 and miR-27a-3p in serum and cerebrospinal fluid of AD patients are consistent. The levels of NEAT1 are increased and the levels of miR-27a-3p are decreased and both are negatively correlated with AD patients that is related to the Aβ degree of sedimentation, which participates in the disease progression of AD and may become a therapeutic target of AD. However, this study only speculates based on the levels of various factors in serum and cerebrospinal fluid of AD patients, which needs to be detected in brain tissue to enhance persuasion. However, it is difficult to obtain brain tissue of AD patients and relevant research will be carried out in AD animal model in the later stage.

## Data Availability

All data generated or analysed during this study are included in this published article.
